# When piloting health services interventions, what predicts real world behaviours? A systematic concept mapping review

**DOI:** 10.1186/s12874-020-00955-7

**Published:** 2020-04-06

**Authors:** Tavis Hayes, Natasha Hudek, Ian D. Graham, Doug Coyle, Jamie C. Brehaut

**Affiliations:** 1 Faculty of Medicine, University of Ottawa, Ottawa, ON Canada; 2 Ottawa Hospital Research Institute, Clinical Epidemiology Program, The Ottawa Hospital, Ottawa, ON Canada; 3 School of Epidemiology and Public Health, University of Ottawa, Ottawa, ON Canada

**Keywords:** Real, Hypothetical, Decision making, Health services, Complex interventions, Systematic concept mapping review

## Abstract

**Background:**

Modeling studies to inform the design of complex health services interventions often involves elements that differ from the intervention’s ultimate real-world use. These “hypothetical” elements include pilot participants, materials, and settings. Understanding the conditions under which studies with “hypothetical” elements can yield valid results would greatly help advance health services research. Our objectives are**:** 1) to conduct a systematic review of the literature to identify factors affecting the relationship between hypothetical decisions and real-world behaviours, and 2) to summarise and organize these factors into a preliminary framework.

**Methods:**

We conducted an electronic database search using PsycINFO and Medline on November 30th, 2015, updated March 7th, 2019. We also conducted a supplemental snowball search on December 9th 2015 and a reverse citation search using Scopus and Web of Science. Studies were eligible to be included in this review if they clearly addressed the consistency between some type of hypothetical decision and a corresponding real decision or behaviour. Two reviewers extracted data using a standardized data collection form developed through an iterative consensus-based process. We extracted basic study information and data about each study’s research area, design, and research question. Quotations from the articles were extracted and summarized into standardized factor statements.

**Results:**

Of the 2444 articles that were screened, 68 articles were included in the review. The articles identified 27 factors that we grouped into 4 categories: decision maker factors, cognitive factors, task factors, and matching factors.

**Conclusions:**

We have summarized a large number of factors that may be relevant when considering whether hypothetical health services pilot work can be expected to yield results that are consistent with real-world behaviours. Our descriptive framework can serve as the basis for organizing future work exploring which factors are most relevant when seeking to develop complex health services interventions.

## Background

In the quest to design new interventions to improve health care, health services research is routinely informed by studies and experiments that incorporate elements different from the real-world application. For example, when designing an intervention to reduce ordering of low-value tests in the ICU, the intervention may not be piloted only on ICU physicians within their day-to-day practice; instead, valid responses are expected to be obtained when data is collected outside of their day-to-day practice, or from non-ICU physicians, or from medical students. A parallel is often drawn with pharmaceutical trials, where prior to definitive trials, considerable preparatory research involves many ‘hypothetical’ elements, including animal models, pilot participants (e.g. patients, clinicians who may differ from the ultimate target group), hypothetical decisions (i.e. would you participate in a study like this?) and pilot settings (e.g. laboratories). The mechanisms studied in this preparatory research are expected to generalize to the ultimate clinical setting, despite these hypothetical or modeled elements, and such preparatory work is considered essential to the overall goal of designing interventions that will work safely and effectively in real clinical settings.

When developing health services interventions, pilot research can incorporate many hypothetical elements. As a multidisciplinary field that studies how personal, organizational, technological, and systemic factors affect access to, quality, and cost of health-care [1], health services research often seeks to design complex interventions [2] to encourage changes in behaviour and decision making among actors (patients, providers, decision makers) within the system. To aid development of these complex interventions, initial work can include piloting decision support tools on healthy volunteers rather than patients, measuring physician performance in simulated settings, and surveying or interviewing people about how they would behave under various hypothetical circumstances.

Despite these tools at our disposal, health services research interventions have often proceeded to large-scale trials without adequate preparatory or pilot research [2–5]. The most recent UK MRC Framework for complex interventions [2] explicitly emphasizes the need to pilot these interventions, in part to model the mechanisms by which one expects the intervention to work before proceeding to large, expensive trials. The reasons why there has been such a lack of preparatory work in health services research are unclear, and may stem in part from a naïve sense of the ease with which such behaviours and decisions can be changed [5, 6]. The study of the mechanisms underlying how health services interventions work is still relatively new [5, 7, 8]. Perhaps as an implicit reaction to the lack of understanding around this issue, there is a disciplinary distrust in pilot data that involve ‘hypothetical’ elements; systematic reviews often exclude studies involving hypothetical elements [9–11] without adequate justification.

We propose that understanding the conditions under which health services studies with ‘hypothetical’ design elements can yield valid results is essential to advancing health services research. With so many elements in these complex interventions, conducting full-scale trials of every permutation is essentially impossible; comparing different combinations in smaller pilot studies with hypothetical elements is inevitable and necessary. While other disciplines (e.g. economics, [12] moral reasoning, [13] social psychology [14]) have explored the conditions under which hypothetical decisions accurately reflect real-world decisions, little of this work has been applied to problems of health services intervention design. As an initial step towards understanding how such factors might be relevant to designing health services interventions, we conducted a systematic concept review of factors that have been shown to be related to the consistency between hypothetical and real-world decisions or behaviours. Based on these findings, we proposed a preliminary framework for those seeking to design a pilot process with hypothetical elements, which summarises and describes factors that may be related to ultimate validity with real-world behaviours.

## Methods

We conducted a systematic concept mapping review, which we define as a review with a systematic search strategy that seeks to delineate the factors related to one or more target concepts; as such, the approach overlaps with systematic reviews and mapping reviews [15]. In this case, we sought to describe and map factors related to ‘consistency’, defined as the association between hypothetical decisions and corresponding real-world decisions or behaviours. In the context of this review, consistency is operationalized liberally as the association between 1) a hypothetical task or pilot task that includes some hypothetical elements, and 2) a corresponding, author-defined ‘real-world’ task, described in the same report. These might include actual real-world tasks or incentivized tasks that the authors claim to represent a ‘real-world’ decision or behaviour. Using the PICO approach to defining studies included in our review, [16] we define our population (P) to include any human study, our interventions (I) to include any factors affecting the relationship between real and/or hypothetical decisions, the comparison (C) to include real vs. hypothetical decisions or behaviours, and the main outcome (O) to be the strength of consistency between those decisions/behaviours.

### Search strategy

We have modeled our reporting on the Preferred Reporting Items for Systematic Reviews and Meta-Analyses (The PRISMA Statement) [17]. Because the core issue has been explored in a variety of research areas, our review was designed to allow us to successfully obtain information from diverse fields. Two of the authors (TH & JB) hand searched the literature to identify a set of target articles that could serve as the foundation for the review. The nine target articles all identified multiple factors that could affect the relationship between hypothetical and real tasks; all were indexed in PsycINFO and/or Medline [18–26]. A health science librarian helped us develop an initial search strategy that included all target articles and involved keyword and titles searches for ‘decision making or behaviour’, ‘hypothetical situations’, and ‘real-world situations’, including synonyms, relevant Medical Subject Headings (MeSH) headings, etc. This search strategy was peer reviewed by a second librarian and modified to develop the final search strategy (see Appendix A). Our search strategy development was guided by the Peer Review of Electronic Search Strategies (PRESS) guideline [27]. We conducted electronic database searches on November 30th, 2015 and March 7th, 2019, a supplemental snowball search on December 9th, 2015, and a reverse citation search using Scopus and Web of Science for studies that cited our target articles.

### Study selection

We conducted a title and abstract screen on all records and liberally included those that might yield factors relevant to the framework; any unclear records were included for further screening. Two of three available reviewers (TH, JB, or NH) independently screened the titles/abstracts for eligibility. The reviewers were not blinded to the journals or authors of the studies screened. To be included in the review, an article needed to clearly address the consistency between some type of hypothetical decision and a corresponding real decision or behaviour. Both empirical and commentary articles were included. Only studies published in English or in French were included. Studies were not excluded based on the setting, time frame, or the date of publication.

After title and abstract screening, the same three reviewers independently screened the full texts of the remaining studies. At this stage, studies were only included if they clearly presented a factor that would be relevant to the framework. The reviewers solved any disagreements through consensus, with JB acting as the final arbiter.

### Data extraction

Three reviewers independently extracted data using a standardized data collection form and the consensus resolution processes described above. This form was developed iteratively during the screening and data collection process. They extracted basic study information (e.g. title, journal, date of publication) and data about each study’s research area, design, and research question. Research area was coded into categories inductively. Design and research question were extracted verbatim from the articles. The type of data supporting the factor was coded as 1) review of multiple articles supporting the relationship (Review); 2) empirical support from a single study or related set of studies (Empirical), or 3) statement or hypothesis without empirical support (Hypothesis). Due to the heterogeneity of the included work in this broad concept mapping review (which included work from many disciplines, as well as empirical, review, and theoretical work), we could not assess the risk of bias in individual studies included in the review, the quality of empirical support underlying each factor, or the risk of bias across studies.

We identified factors presented in the study by selecting quotes that named and described the relevant factor. Two coders (TH and NH) extracted the quotes from each study to describe how the factor affected the consistency between hypothetical and real decisions. These quotations were then summarized to produce initial factor statements. A third person (JB) supervised and corroborated this coding.

### Data analysis and framework development

Our approach to data analysis resembled what Hsieh & Shannon (2005) call a “Conventional Content Analysis.” [28] This inductive approach is useful when existing theory around a phenomenon being described is limited [28]. Based on the extracted study quotations and initial factor statements, we developed standardised statements describing each factor in terms of whether it was predicted to increase or decrease consistency. The coders then made collaborative decisions about when similar concepts were combined into a single factor. Where possible, we used the authors’ own descriptions of the concepts to make these decisions.

As part of a preliminary framework development process intended to summarize and categorise the factor statements [29], raters made initial attempts at organizing the different factors into categories. After discussion yielded a mutually agreed upon set of categories that were thought to be largely mutually exclusive and potentially useful in thinking about how to design model studies, two coders (TH and JB) independently assigned each factor to a category; discussion resolved any conflicts. In situations where the sign of the association with consistency depended largely on phrasing (e.g. a positive association between consistency and ‘certainty’ might have been coded as a negative association between consistency and ‘uncertainty’), coding was decided based on clarity and the manner of presentation in the original articles.

## Results

**Fig. 1:**
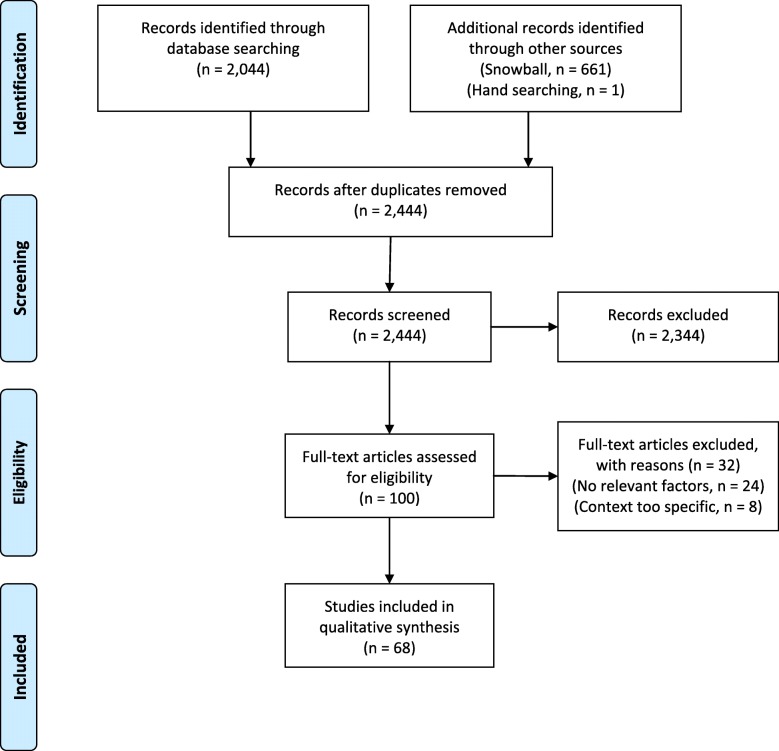
PRISMA flow diagram. From: Moher D, Liberati A, Tetzlaff J, Altman DG, The PRISMA Group. Preferred Reporting Items for Systematic Reviews and Meta-Analyses: The PRISMA Statement. PLoS Med. 2009;6(7):e1000097. doi:10.1371/journal.pmed1000097

Figure 1 describes the PRISMA flow diagram for our concept review. After duplicates were removed, the abstracts of 2444 articles were screened; 2344 of these were screened out as unrelated to the topic of consistency between real and hypothetical decisions or behaviours, or not published in English or French. The remaining 100 articles underwent full text screening; 24 were excluded for lack of any identifiable factor relating hypothetical and real-world decisions or behaviours, while another 8 were identified as being too ‘context-specific’, meaning they described factors that likely had limited application to health services interventions (e.g. ‘intention to conduct criminal acts’), or because they were unrelated to consistency. The remaining 68 articles came from a range of literatures, including behavioural economics (44 articles), the psychology of reasoning/behaviour (14 articles), social psychology (7 articles), health behaviours (4 articles), and neuroscience (5 articles). The 68 articles identified 27 factors purported to modify the relationship between hypothetical and real-world decision making. For details on the included articles see Appendix B. Our consensus process identified 4 categories of factors as described below. Tables 1, 2, 3 and 4 correspond to these 4 categories, and provide name and definition of the factor, its proposed specific relationship to consistency, type of data supporting the relationship, and corresponding citations.

**Table 1:** Decision maker factors

Factor	Definition	Association with consistency	Type of supporting data
Age	Age of the decision-maker	Older decision makers show more consistency between hypothetical and real life decisions	Empirical [30]
Education	Educational attainment of the decision-maker	More educated decision makers show more consistency between hypothetical and real decisions	Empirical [30]
Cognitive Control	The decision-makers use of mental processes to concentrate and think	Higher cognitive control is correlated to inconsistency between hypothetical decisions and real decisions	Empirical [23]
Cognitive Ability	Cognitive ability of the decision-maker	Higher cognitive ability increases risk aversion for hypothetical decisions but not for real life ones	Empirical [25]
Thinking Dispositions	Whether decision-makers have dispositions about thinking that allow them to accept strategies to make hypothetical reasoning resemble real-world reasoning	Being in a state of prolonged doubt increases correlation between hypothetical and real life decisions	Hypothesis [21]
Openness to Experience	Measure of the decision-maker’s openness to a variety of ideas and experiences	Greater openness to experience trait (IASR-B5) is predictive of hypothetical decisions but not real ones	Empirical [31, 32]
Other Personality Traits	Personality traits, other than openness to experience, of the decision-maker	Personality traits, especially extraversion low neuroticism, and anti-social traits correlated with inconsistently between hypothetical and real life decisions	Empirical [13, 32–34]

**Table 2:** Cognitive factors

Factor	Definition	Association with consistency	Type of supporting data
Normative Beliefs	Whether the decision-maker is thinking about what important others would think about their decision	Normative Beliefs are less likely to be activated for hypothetical decisions than for real ones	Empirical [35]
Social Desirability	Whether the decision-maker’s decision is affected by their desire to conform to the experimenter’s beliefs	Social desirability affects hypothetical decisions more than real decisions	Review [36, 37] Hypothesis [13, 38]
Anticipated or Forecasted Emotions	Whether people are predicting the emotions they think they would feel when making a decision, versus actually experiencing those emotions	Emotions in hypothetical decisions are forecasted more than in real life decisions	Review [14, 37, 39] Empirical [19, 40–42]
Deliberative Mindset	Whether participants are evaluating the pros and cons of different options, versus focussing on information that is useful for them to complete a selected goal	The deliberative mindset is used more in hypothetical decisions than in real life	Review [14]
Abstract Construals	Whether the decision-maker is thinking about the general features, versus thinking about the specifics of a decision	Abstract construals of problems are employed more in hypothetical decisions than in real life	Review [14] Empirical [43]
Attribute Non-Attendance	Whether the decision-maker neglects to fully consider some of the attributes of a decision	More attention is paid to the attributes of a real decision than a hypothetical one	Empirical [44]
Risk Aversion	Whether the decision-maker prefers options that are less likely, but have greater rewards, to options that are definite but have smaller rewards	Risk aversion is underestimated in hypothetical decisions compared to real life ones.	Empirical [24, 45–48]
Implicit Associations	Amount of automatic associations elicited in the decision	Consistency between real and hypothetical decisions is worse with more implicit associations present	Empirical [49]
Certainty	Whether the decision-maker is certain that their hypothetical decision is the same as would be their real-world decision	A high degree of certainty about a hypothetical decision makes it more likely to be consistent with a real decision	Review [50–52] Empirical [26, 53–55]
Salience of/ Concern with the Decision	Amount of importance placed on hypothetical decision	Greater engagement/concern associated with greater consistency	Empirical [20, 22, 31, 56–59]

**Table 3:** Task factors

Factor	Definition	Association with consistency	Type of supporting data
High Stakes Rewards	The size of rewards/incentives being offered	When large incentives are available, risk aversion is higher for real life decisions than for hypothetical decisions	Review [60, 61]
Framing Effect	Whether the decision is framed in a way that is positive (i.e. gains) or negative (i.e. losses)	The framing effect may be larger for hypothetical decisions than for real life ones	Empirical [62]
Explicit Statements of Uncertainty of Outcomes	When estimates of the probability of the outcome are explicitly presented to the decision-maker	Providing statements about uncertainty increases consistency between hypothetical decisions and real life	Review [60]
Fundamental Attribution Error	Whether the decision is worded in a way that asks the decision-maker what they would do or asks what they think someone else should do	Presenting the hypothetical decision with the decision-maker as the actor (as opposed to an observer) increases consistency between hypothetical and real-world decisions	Empirical [63]
Personal Relevance	Whether the decision being made is one that involves people with whom the decision-maker has long-term relationships	Personal relevance of a problem is correlated with consistency between hypothetical and real decisions	Empirical [64]
Real Consequences	Whether the decision has real consequences for the decision-maker	Having real consequences makes hypothetical decisions more closely predict real-world ones	Review [37, 51, 52, 65–67] Empirical [43, 47, 67–69]
Space for Mental Simulation	The degree to which the context of the decision is left to the imagination	Greater space for mental simulation associated with lower consistency	Empirical [18, 70]
Self-Image	Whether the decision relates to the decision-maker’s self-image (e.g. related to their ethical beliefs)	Decisions related to self-image show less consistency between hypothetical and real-world decisions	Review [51, 71] Empirical [72]

**Table 4:** Matching hypothetical and real-world tasks

Factor	Definition	Association with consistency	Type of supporting data
Matching Samples	Whether the sample of people making the hypothetical decision closely resembles the population that faces the real-world decision	When participants in hypothetical situations resemble the target real-world group, hypothetical decisions are more consistent with real-world ones	Empirical [56, 73–75]
Matching Procedures	Whether the study procedures (e.g. what decision is being made and how the information is presented) for both hypothetical and real tasks are matched.	When the procedural characteristics of a hypothetical decision resemble the real-world decision, consistency will be higher	Review [12, 37, 39, 65, 76] Empirical [69, 74, 77–85]

### Decision maker factors

Decision maker factors are those traits/capacities that relate directly to the decision maker themselves. Table 1 describes seven factors of the decision maker studied in relation to the extent to which hypothetical decisions will match real-world decisions/behaviours. Relatively little data supported an association with basic demographic factors; for example, we were unable to find any clear associations with *sex* or *ethnicity*; however, one study reported possible gender differences in their results [38]. More convincingly, another study reported greater consistency in willingness to pay donation decisions with

1. *Greater age* of the decision maker, and
2. *Higher education* of the decision maker, both in the context of willingness to pay decisions [30].

More work has explored the extent to which capacities of the decision maker affect consistency, including

3) *Cognitive control* (higher cognitive control associated with lower consistency), and
4) *Cognitive ability* (higher scores showing lower consistency). Both were based on EEG studies involving participants choosing between hypothetical or real lottery options [23, 25]. In these studies, those with greater cognitive capacity or control were hypothesized to incorporate a greater number of issues into their decision making, considerations that made them less risk averse in hypothetical situations than in real situations.
5) *Thinking dispositions* (e.g. enjoy challenging ideas), where one study argued that such dispositions are related to greater consistency [21].

Several studies also explored apparently complex relationships between personality traits and consistency, including

6) *Openness to experience,* where higher openness may be negatively related to consistency in the context of moral cooperation decisions; openness to experience was predictive of real (incentivized) decisions, but not hypothetical decisions [31, 32].
7) *Neuroticism, agency, and anti-social attitudes,* where traits have been explored in their association with inconsistency across real-world and hypothetical decisions [13, 32, 33].

### Cognitive factors

Cognitive factors are characteristics related to the decision-making process. Table 2 describes the ten cognitive factors identified as related to consistency. Several factors suggested negative associations, including activation of

1. *Normative beliefs*, where real donation decisions were affected by consideration of what important others (e.g. family members) would think of their decisions in a way that hypothetical decisions were not [35];
2. *Social desirability*, where a review of the literature shows that the wish to be seen favourably by the experimenter is stronger for hypothetical than real-world decisions [36];
3. *Anticipated or forecasted emotions*, given the extensive literature that shows that people are poor at predicting how they will feel in the future; similar issues are discussed under related terms such as ‘hot-cold empathy gap’, [19, 40] or ‘predicted vs expected utility’ [39];
4. *Deliberative mindset*, where individuals making hypothetical decisions may be more likely to carefully weigh pros and cons than those making real-world decisions [14];
5. *Abstract construals*, where hypothetical decisions are more likely to involve consideration of general vs specific features of the decision [14];
6. *Attribute non-attendance*, where decision makers are more likely to consider all relevant attributes in real-world than hypothetical decisions [44];
7. *Risk aversion*, where decision makers are often more likely to choose safer courses of action in real-world as compared to hypothetical situations [24, 45, 46];
8. *Implicit associations,* where a greater amount of automatic associations related to less consistency [49].

Our review also identified factors of cognition that suggest positive associations with consistency, including

9) *Certainty*, where decision makers who are more certain of their hypothetical decisions are more likely to be consistent with real-world decisions [25, 50, 53, 54];
10) *Salience of or concern about the task,* where increasing salience of the decision or task (e.g. by increasing incentives, making the task more interesting, ensuring self-benefit, etc.) can increase consistency [20, 22, 31, 56, 57].

### Task factors

Task factors include aspects of the hypothetical decision being made, independent of the match with the real world decision scenario. Table 3 describes the eight characteristics of the hypothetical task identified as related to consistency. Factors include

1. *High-stakes rewards;* two reviews of the literature have pointed to high stakes decisions as being negatively associated with consistency- the higher the stakes, the lower the association between hypothetical and real [60, 61].
2. *Framing bias* (i.e. biases in decisions produced by providing outcome probability statements in terms of positive vs. negative frames) showing that this effect is more powerful for hypothetical than real-world decisions, reducing consistency [62].
3. *Explicit Statements of uncertainty of outcomes*, where having explicit statements describing the range of uncertainty around outcome estimates in the hypothetical task has been shown to be positively associated with consistency [60].
4. *Fundamental attribution errors,* where describing the decision maker as the direct actor, as opposed to an observer in the hypothetical task may be positively associated with consistency [63].
5. *Personal relevance,* where ensuring that the hypothetical task involves people the decision maker actually knows may be positively associated with consistency [64].
6. *Real consequences,* where ensuring that the hypothetical task entails actual consequences for decision makers is positively associated with consistency [51, 68].
7. *Space for mental simulation* (i.e. the degree to which the context of decision making is left to the imagination) may be associated with lower consistency [18, 70].
8. *Self-image,* where several studies have explored the notion that moral decisions may have lower consistency, given the tendency to preserve a positive view of oneself (i.e. more likely to make positive choices in hypothetical decisions than in real life) [51, 71, 72].

### Matching hypothetical and real-world tasks

Table 4 describes two related issues identified as increasing consistency by matching the hypothetical and real-world in different ways. These literatures discussed issues of consistency less directly, and as such coders were less able to identify specific tests of the relationship between consistency and individual factors. Coders felt that these issues were core to the issue of consistency despite the lack of explicit relationships, hence the inclusion of these issues.

1. *Matching samples* with the real-world population has been discussed extensively in various literatures. Many have argued that representative samples are essential in increasing consistency (e.g. Hainmueller et al., 2015, Kesternich et al., 2013 [56, 73]) and an extensive literature has explored the extent to which specific types of samples yield generalizable results (e.g. Berinsky et al., 2012, Peterson et al., 2014 [86, 87]). One study examining the validity of different survey designs in determining immigrant acceptance decisions demonstrated that samples that demographically reflected the target group matched real-world decisions more closely than did a sample of students [56]. Reviews of the extensive literature on the use of college students as subjects in social science experiments have shown that student samples often do not yield results that are reproducible in broader populations [61, 88]. Note that we did not find any studies that sought to describe what patient characteristics need to be matched in order to ensure validity with a real-world health study.
2. *Matching study procedures* to the real-world decision contexts has also been explored extensively. Studies varying apparently minor deviations of the hypothetical decision-making context (e.g. number of cues, order of presentation) have often shown effects on complex decisions; matching on as many of these cues as possible has been argued to increase consistency [76]. For example, considerable work has examined delay discounting, i.e. the rate at which a good (or a health benefit) decreases in value depending on the amount of delay in receiving it. Chapman (2004) [39] discusses discounting in the context of health behaviours, like addiction. While most agree [69, 77] that the rate of delay discounting is generally consistent between hypothetical and real-world situations, [39, 77–83] matching the decision-reward delay between hypothetical and real decisions improves consistency even further [84]. In a study of children’s reactions to social problems, authors argued that having more time to decide in the hypothetical than the real situation would reduce consistency [85]. Other study authors have argued that matching contextual features of the hypothetical task to the real-world decision as closely as possible is essential for generalizable results [69, 89]. This concept has been taken one step further, where authors argue the overall complexity of the decision environment in real-life situations becomes oversimplified in hypothetical choices, leading to poor choice consistency [74].

## Discussion

If the health services research community is to systematically implement recommendations for better modelling prior to large scale interventions, [90] we need to understand how health care decisions and behaviours can most effectively be modelled. Given that most health service interventions seek to change the decisions or behaviours of different actors within the system (e.g. physician test ordering, patient participation decisions), we must design model studies in which hypothetical decisions/behaviours can be valid indicators of their real-world counterparts. In this review, we sought to summarize what is known about factors thought to affect the relationship between hypothetical and real-world decisions. Our review of 68 articles identified 27 factors shown or hypothesized to affect the relationship between hypothetical and real-world decisions/behaviours. Coming from a wide range of literatures, including behavioural economics, psychology of reasoning, social psychology, health behaviours, and neuroscience, these findings clearly underline the fact that much is already known about how to help decisions and behaviours made in hypothetical contexts reflect real world decisions. Equally clear is that relatively little of this discussion has focused on health behaviours (4 of 68 articles), further underlining the need to explore these issues for health decisions.

**Fig. 2:**
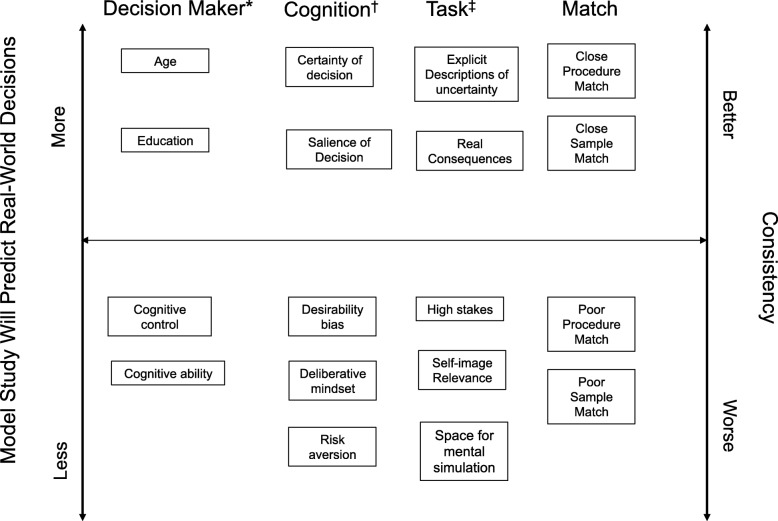
Descriptive framework of the 4 categories of factors identified as related to consistency. ^*^Decision maker category also includes thinking disposition, openness to experience, and other personality traits. ^†^Cognition category also includes normative beliefs, forecasted emotions, abstract construals, attribute non-attendance, and implicit associations. ^‡^Task category also includes framing effect, fundamental attribution error, and personal relevance

Figure 2 summarizes our descriptive framework of the four categories of factors identified to be related to consistency; i.e. whether hypothetical decisions will predict real-world behaviours. Above the center line are examples from each category that are positively associated with consistency; below the line indicates negative associations. *Decision maker factors* include specific trait-level descriptors that vary between (but usually not within) individuals, and may be positively (e.g. age, education) or negatively (e.g. cognitive ability) associated with consistency between hypothetical and real decisions/behaviours. *Cognitive factors* describe internal, context-dependent factors (e.g. certainty, risk aversion) that may affect human decision making in general, but are particularly relevant to hypothetical-real consistency. *Task factors* include important aspects of the hypothetical task (e.g. describes the uncertainty of outcomes, involves real consequences) that are related to consistency independent of their relationship to the real-world task. Finally, *matching factors* identify areas where an overall increase in similarity between the model situation and the real-world (sample matching, procedure matching) would be expected to improve consistency; a more fine-grained analysis of these two categories will be required to identify specific factors within the context of overall complexity of the environment.

We offer this draft framework not as a recipe for optimal design of model health care studies, but as a way of organizing and describing the range of factors that might need to be explored to achieve this end. The extent to which any individual factor will predict consistency in the context of health services decisions/behaviours is almost entirely open to debate at this early stage. Few of these factors have been tested in a health services context (but see Appendix B for examples of matching procedures, [39, 65, 81] real consequences, [65] degree of certainty, [53] and forecasting emotions [39]). The potential for interactions between factors in affecting consistency is almost entirely unexplored. The data supporting them at all are highly variable, ranging from extensive literatures summarized by systematic review to suppositions made without any empirical support. For this initial description, we chose to include all factors regardless of the level of empirical support or potential for bias in order to provide the greatest range of hypotheses to consider as we push this area forward.

Several limitations of this work warrant consideration. First, while our search strategy sought to encompass as many synonyms for ‘hypothetical’ and ‘real-world’ decisions as possible, there are likely studies touching on this issue that were not captured by our search. For example, our search strategy did not include keywords specific to simulation teaching methods in the healthcare field. While the consistency between real and hypothetical decisions is relevant to the medical education field, that literature focuses on methods to help students make the ‘right’ decision (e.g. how objective structured clinical exams predict correct medical decisions). In contrast, our review focused on aspects of hypothetical decisions and their consistency with a real world decision independent of its ‘correctness’. Second, many of the included studies from the behavioural economics literature involved the common practice of using incentives to distinguish hypothetical vs real-world decisions; a ‘real-world’ task implied one where participants were incentivized with tangible rewards, while hypothetical tasks involved no incentives. Although using incentives is known to increase motivation for a range of health behaviours, [91, 92] we do not know the extent to which simple incentives can serve as a model for complex, high-stakes, often emotion-laden health care decisions. On a related note, for this initial multi-discipline concept review, we could not assess the degree to which ‘real-world’ tasks were ‘real’ enough; instead, we took the authors’ word that providing a $5 incentive (for example) was an effective approach for modeling real-world decisions. Third, our initial framework is meant to be descriptive and does not attempt to identify relative importance of the described factors, or the causal relationships and interactions between them (as it does not constitute a theory). Fourth, we cannot make strong claims about the strength of the data underlying any particular factor and its relationship with consistency; while we sought to distinguish factors supported by considerable empirical support vs those without, a stronger assessment of the quality of evidence supporting the individual relationships, and the risk of bias associated with these varied studies, was beyond our resources. Therefore, as new research becomes available, future work should focus on a meta-analytic review of empirical studies to evaluate the risk of bias for the factors we have identified, as well as establishing statistical significance of these factors in predicting the consistency between real and hypothetical decisions and behaviours. Finally, we note that some of the identified factors (e.g. forecasting emotions, matching sample factors) are supported by substantial literatures and considerable theoretical discussion that provide a level of nuance we could not address in this review. The implications these non-health literatures have for health services research applications is a clear area of future work.

## Conclusions

This review identifies a range of factors that may be relevant in determining when hypothetical pilot work can be expected to yield results that are consistent with real-world health services behaviours. We have highlighted four categories that appear to encompass these factors, categories that may be helpful to consider for those designing pilot health services work. Future work can use our list of factors as the range of hypotheses that must be tested to determine which factors are most important in determining consistency in a health services context. In health services research, it is rare that hypothetical work is reported in the same article with real-world trial results. Compiling health services research programs where hypothetical pilot work can be matched to reports of real-world outcomes would be a useful step in understanding when and how to maximize the utility of hypothetical health services research.

## Data Availability

The data generated and used during the current study are available from the corresponding author on request.

## Appendix A

### Search Strategy for PsycINFO and OVID MEDLINE (R) ALL

**Terms for decision making or behaviour:**

1. Decision Making/ (156670).

2. Choice Behavior/(48,223).

3. reasoning.ti,ab. [No MeSH term] (56346).

4. Behavior/ (53175).

5. (decision* or choos* or choice* or behavio?r*).ti,ab. (2851118).

6. Risk-Taking/ (37079).

7. (tak* adj2 risk*).ti,ab. (19838).

8. or/1–7 (2965585).

**Terms for hypothetical situations:**

9. Uncertainty/ (18393)

10. hypothetical*.ti,ab. (47294).

11. proxy.ti,ab. (25748).

12. (formal adj3 (reasoning or thinking or decision*)).Ti,ab. (1459)

13. or/9–12 (92440).

**Terms for real world situations:**

14. Reality/ (4401).

15. (real or reality).ti,ab. (554361).

16. everyday.ti,ab. (74373).

17. or/14–16 (623798).

18. 8 and 13 and 17 (2044)

19. remove duplicates from 18 (1782)

## Appendix B

**Table 5:** Table of papers included in the review, with basic details including research area, design, research question, type of data, and factor(s) identified

Ref. #	Author	Year	Research Area	Design^a^	Research Question^a^	Type of Data	Factor
[35]	Ajzen	2004	Behavioural economics	Respondents to a survey were asked their willingness to pay for a certain good (contribute to a scholarship fund) in a hypothetical or contingent market.	The study explored the reasons for hypothetical bias. Secondary aim was examining the effect of a corrective entreaty on bias.	Empirical	Normative beliefs
[66]	Anselme	2015	Behavioural economics	Review argues that risk is only present if the consequences affect on resources (lack of reward is not enough).	The occasional and unpredictable absence of reward is a negative consequence interpreted as risk.	Review	Real consequences
[43]	Barkan	2016	Behavioural economics	Five experiments where participants were randomly assigned the role of chooser (treated as real choice) or adviser (treated as hypothetical choice). Examined difference between behaviours when acting as a chooser vs. adviser.	Hypothesize choosers will experience more curiosity than they predict others will have as advisers. Choosers will purchase the costly and useless information, while acting as advisers they will recommend against this action. In hypothetical situation chooser and adviser behaviour will be more similar.	Empirical	Real consequences; abstract construals
[58]	Beattie	1997	Behavioural economics	Common ratio effect, anticipated regret, behaviour toward a particular form of multi-stage gamble. Hypothetical, random problem selection procedure, and real settings.	How far and in what ways do incentives, or lack of incentives, influence responses.	Empirical	Salience of/ concern with the decision
[81]	Bickel	2010	Behavioural economics; health	Hypothetical delay discounting task and possibility to earn vouchers for consecutive negative urine analysis.	They examined delay discounting measures in predicting whether an opioid-dependent earning voucher in a clinical trial would be redeemed frequently or not, and if delay discounting predicted the voucher redemption rates.	Empirical	Matching procedures
[53]	Blumenschein	2001	Behavioural economics; health	Both hypothetical and real groups received a valuation question about their willingness to pay for an asthma management program. If they said yes, they were asked about their degree of certainty. The real group could purchase the program.	They conducted a field experiment of hypothetical versus real willingness to pay for a health care good.	Empirical	Certainty
[55]	Blumenschein	1998	Behavioural economics	Hypothetical then real, or only real; question about purchasing sunglasses. Hypothetical question was followed by probably sure or definitely sure. Real group offered option to purchase.	An experiment comparing the dichotomous choice contingent valuation method with real decisions for a consumer good.	Empirical	Certainty
[13]	Bostyn	2018	Moral reasoning	Two samples from the same student population: a group of students completed a real-life version of the mouse dilemma, while a second group completed a hypothetical version of the same dilemma.	They studied differences in the classic trolley dilemma using real mice receiving a shock. Examined anti-social personality traits in relation to hypothetical and real choice.	Empirical; hypothesis	Personality traits; social desirability
[36]	Camerer	1999	Behavioural economics	Review of 74 studies in relation to financial incentives.	They summarize the results of 74 studies comparing behavior of experimental subjects who were paid zero, low or high financial incentives in both real and hypothetical studies.	Review	Social desirability
[37]	Camerer	2017	Neuroscience	Review compares evidence of mental processes during real and hypothetical choices.	They evaluate evidence of differences in hypothetical and real behavior and brain activity in five areas: social, moral, emotional, economic choice, and vision.	Review	Real consequences; matching procedures; social desirability; emotional forecasting
[38]	Ceccato	2018	Social psychology	They investigate the relationship between perceived stress and social preferences in a 2x2x2x2 anonymous dictator game. They manipulated the gender of the sender and recipient, the frame (give vs. take), and the nature of the reward (real or hypothetical money).	They hypothesize that chronic stress is positively related to both real and hypothetical money transfers.	Hypothesis	Social desirability
[39]	Chapman	2004	Behavioural psychology; health psychology	Summary of literature related to the psychology of medical decision making in six areas.	One area reviewed was whether decisions in hypothetical questionnaire scenarios are related to real-world health behavior.	Review	Emotional forecasting; matching procedures
[31]	Day	1998	Behavioural psychology	Standardized measures of personality and Kohlberg’s moral maturity. Asked to distribute money among 4 people. Relevant groups: hypothetical, real people fake money (play), and real.	The goals of the study were to evaluate aspects of Kohlberg’s model, and to examine one alternative—the interaction additive/inclusive model.	Empirical	Openness to experience; salience of/ concern with the decision
[84]	Dixon	2013	Behavioural psychology	Participants made choices between varying real amounts of money and a fixed delayed amount.	The study examined whether actual and hypothetical delays have similar effects on delay discounting. Also compared the discounting of hypothetical and real monetary rewards.	Empirical	Matching procedures
[14]	Eastwick	2013	Social psychology	Review of three perspectives that may help determine when a study will be externally valid.	They draw from existing psychological theories to predict differences between laboratory research and externally valid, field-like research.	Review	Abstract construals; deliberative mindset; emotional forecasting
[76]	Ebbesen	1980	Behavioural psychology	Review and discussion of studies comparing decision making in the laboratory to decision making in real life in four areas: bail setting, sentencing of adult felons, automobile driver behavior, and judging swine.	They state that many current models of decision making are based on evidence from laboratory experiments where a limited set of simulated decision problems have been used.	Review	Matching procedures
[57]	Etchart-Vincent	2011	Behavioural economics	Binary lottery versus sure amount. Conditions included: real losses, hypothetical losses, losses from endowment, real versus hypothetical gains.	The study aimed to systematically explore whether subjects’ risk aversion over losses depends on the payment scheme, including real vs. hypothetical gains.	Empirical	Salience of/ concern with the decision
[20]	FeldmanHall	2012	Social psychology; neuroscience	Subjects were asked about their willingness to receive money by causing pain to another subject in hypothetical and real (but actually fake) settings. Used fMRI to see whether the same neural areas were activated.	They hypothesized that their moral conflict would provide an ideal method to examine the behavioral and neural differences between intentions and actions.	Empirical	Salience of / concern with the decision
[18]	FeldmanHall	2012	Behavioural psychology	Two studies where they compared decisions in real and hypothetical conditions by asking participants whether they would be willing to spend money to prevent harm to another person. The second study increased the richness of hypothetical contextual cues.	They aimed to investigate moral decision-making in situations where harm to another person and personal gain act in opposition, and examine how this moral tension was resolved in hypothetical and real contexts.	Empirical	Space for mental simulation
[21]	Galotti	1989	Reasoning	Literature review examining three approaches to the study of reasoning that extend beyond one specific task: the componential approach, the rules/heuristics approach, and the mental models/ search approach.	Purpose is to assess the strengths and weaknesses of three approaches in accounting for performance in a variety of contexts and on a variety of tasks, both laboratory and every day.	Hypothesis	Thinking dispositions
[75]	Gold	2014	Behavioural psychology	Two studies using real and hypothetical variants of the trolley dilemma. Compared British to Chinese participants	They operationalize a version of the trolley problem in which the harms are small but meaningful economic losses, and compare the actual and hypothetical choice behavior of British and Chinese samples.	Empirical	Matching samples
[63]	Gold	2015	Behavioural psychology	2 × 2 design. Footbridge vs. Side-track and actor vs observer. Actors made decisions that influenced the amount of money to donate to an orphanage in Northern Uganda and observers told the investigators what decision the actor should take.	They investigated whether there were behavioral differences between different trolley problems, what the patterns of moral judgments were in real-life trolley problems, and if behavior corresponds to moral judgments.	Empirical	Fundamental attribution error
[33]	Grebitus	2013	Behavioural economics	2 × 2 design. Hypothetical and non-hypothetical choices. Products were apples and wine.	They examined whether personality predicts behavior in hypothetical and non-hypothetical choice experiments and auctions.	Empirical	Personality traits
[56]	Hainmueller	2015	Social psychology	Compared five different vignette types asking participants about whether they would accept the potential immigrant described. Compared results to real-world naturalization referendums.	They examined whether a survey experimental design would be similar to the behavioral benchmark and if there was important variation in the performance of the various designs.	Empirical	Salience of/ concern with the decision; matching samples
[60]	Harrison	2005	Behavioural economics	Review of studies that considered hypothetical bias over uncertain outcomes.	Paper reviews evidence for whether estimates of risk attitudes defined over monetary outcomes suffer from hypothetical bias.	Review	Explicit statements of uncertainty; high stakes rewards
[52]	Harrison	2008	Behavioural economics	Book chapter reviews studies that considered hypothetical bias in value elicitation methods.	They review experimental results that support hypothetical valuation exceeding real valuation. They examine two bodies of literature.	Review	Certainty; real consequences
[68]	Hinvest	2010	Behavioural economics	Delay discounting: Eight blocks of 30 binary choice trials (4 real, 4 hypothetical) with probability discounting (wheel of fortune).	They explored the effect of real versus hypothetical reward on choice behavior using real-time delay discounting and probability discounting tasks.	Empirical	Real consequences
[45]	Holt	2002	Behavioural economics	Lottery choices. Participants were presented with paired lottery choices in real payoff and hypothetical conditions.	They present subjects with simple choice tasks to estimate the degree of risk aversion and specific functional forms.	Empirical	Risk aversion
[46]	Holt	2005	Behavioural economics	Lottery choices. Two groups: 1. Real low-payment followed by real high-payment. Second group only did one lottery choice menu (low/high-real/hypo)	They replicate finding that the order effect (participating in a low-payment choice before making a high-payment choice) magnifies the scale effect. Then eliminate the order effect in a subsequent study.	Empirical	Risk aversion
[22]	Irwin	1992	Behavioural economics	Vickrey auction; 1% chance of a $40.00 loss. Hypothetical and real money groups. Length of experiment was varied to test for effects of boredom.	They presented subjects with an objective risk, over a number of trials, with feedback and consequences dependent on behavior to test the effect of reward type only. They also varied the task length, to see if boredom effects of hypothetical rewards became pronounced in longer experiments.	Empirical	Salience of/ concern with the decision
[41]	Joel	2015	Social psychology	Two studies. Single participants were given the option to accept or reject a potential date in what they believed to be either a hypothetical or a real-life context.	They hypothesized that people making decisions about whether to accept or reject a potential romantic partner are influenced by their desire to avoid causing that person harm, and that people underestimate this source of influence.	Empirical	Emotional forecasting
[54]	Johannesson	1999	Behavioural economics	Data from dichotomous choice experiments, real and hypothetical (chocolates and sunglasses).	They present a method for identifying a subset for which hypothetical yes responses represent real yes responses.	Empirical	Certainty
[72]	Johansson-Stenman	2012	Behavioural economics	Choice experiment using real and hypothetical decisions for moral (donation to WWF) and amoral (restaurant voucher) goods, selected at random to be replicated in a real setting.	They develop and test a theoretical model aimed at explaining variations of hypothetical bias in other studies.	Empirical	Self-image
[74]	Johnson	2018	Social psychology	Three studies testing how dispatch information and police experience impact the decision to shoot. Compared decisions of police with students.	They used the drift diffusion model to outline different mechanisms by which race, dispatch information, and police experience could impact the decision to shoot.	Empirical	Matching procedures; matching samples
[78]	Johnson	2002	Behavioural economics	Within subject measure of delay discounting for hypothetical and (potentially) real rewards. Monetary rewards, greater magnitudes than previously used in the literature.	They measured delay discounting of real and hypothetical rewards using both exponential and hyperbolic decay models to describe the data.	Empirical	Matching procedures
[40]	Kang	2013	Behavioural economics	Fifty aversive food items. Rated familiarity with foods, bid to purchase the right not to eat the food in hypothetical and real contexts.	Their goal was to see if there was distinct neural valuation during hypothetical and real choices.	Empirical	Emotional forecasting
[73]	Kesternich	2013	Behavioural economics	Online survey asking to older participants to choose between insurance contracts and compared to real choices. Varied prices.	They investigated whether hypothetical choice experiments can answer questions during the design phase of a program.	Empirical	Matching samples
[67]	Klein	2019	Behavioural economics	Review: raters classified studies into fully consequential choice, partially consequential choice, hypothetical choice, and non-choice task. Experiment: compares the first three choice classes in the same setting.	They tested for hypothetical bias in fully consequential, partially consequential, and hypothetical choices to understand whether having the possibility to consume the product alters choices.	Review; empirical	Real consequences
[19]	Kuhberger	2002	Behavioural economics	Two studies using a gambling paradigm. Varied positive and negative framing in real and hypothetical conditions.	Examined specific criteria where hypothetical situations can be used instead of real ones.	Empirical	Emotional forecasting
[79]	Lagorio	2005	Behavioural economics	Participant presented with both real and hypothetical rewards to purchase snacks from the researchers, both immediately and after a delay.	Examined delay discounting of real and hypothetical consumables.	Empirical	Matching procedures
[80]	Lawyer	2011	Behavioural economics	Compared probability and delay discounting rates and patterns of non-systematic response in hypothetical and potentially real conditions with non-substance abusing and substance dependent individuals.	The purpose of the study was to compare patterns of delay and probability discounting in two samples (substance dependent and not dependent).	Empirical	Matching procedures
[62]	Levin	1988	Behavioural economics	Gambling task, asked to evaluate gambles based on likelihood of picking a task and confidence in that judgment. Probability and/or payoff information was given. Positive and negative conditions. Hypothetical and Real.	The goals the study were to: (1) to examine how confidence in judgments is affected by the absence of information; (2) to compare conditions where subjects are or are not required to make explicit inferences; (3) to replicate and extend earlier results of frame effects; and (4) to examine the external validity in a real context.	Empirical	Framing effect
[71]	List	2001	Behavioural economics	Meta-analysis examining evidence pertaining to the effects of various experimental protocols on the calibration factors related to hypothetical bias.	They examine various questions related to hypothetical bias, contingent valuation, willingness-to-pay/accept, elicitation methods, benefits of within-subjects designs, lab vs field experiments, and public vs private goods.	Review	Self-image
[50]	Little	2004	Behavioural economics	Meta-analysis examining conditions that influence discrepancies between real and stated values.	They updated and expanded a meta-analysis to identify the conditions that may influence the disparity between actual and stated values.	Review	Certainty
[32]	Lonnqvist	2011	Behavioural economics	Incentivized and hypothetical prisoner’s dilemma game.	They investigated the effect of incentives on research outcomes, by focusing on the big five personality determinants of incentivized or hypothetical behaviour in the prisoner’s dilemma game.	Empirical	Openness to experience; personality traits
[77]	Madden	2003	Behavioural economics	Participants were quasi-randomly assigned to complete a real or hypothetical reward system first. They chose between immediate or delayed rewards.	They sought to further explore the relation between reward type and rates of delay discounting.	Empirical	Matching procedures
[82]	Madden	2004	Behavioural economics	Two experiments: (1) real rewards group given the amount they selected and hypothetical group received a flat rate. Choose between immediate and delayed rewards, and (2) with fewer choices to increase the proportion of real choices.	They sought to compare discounting rates when potentially real and hypothetical rewards were used in both between- and within-subjects methods.	Empirical	Matching procedures
[30]	Mjelde	2012	Behavioural economics	Analysed data from three previously existing studies. Studies assessed people’s hypothetical and real willingness to pay by asking them if they were willing to make a donation.	The aim of the study was to (1) examine the relationship between discounting of real and hypothetical rewards; and (2) to examine the one-week test-retest reliability of these rewards.	Empirical	Age; education
[12]	Morales	2017	Behavioural economics	Review paper looking at the the choice of independent variables along the experimental-realism dimension (artificial to real) and the choice of dependent variables along the behavioral-measures dimension (hypothetical intention to actual behavior).	They examined the importance and benefits of using realistic manipulations and measuring actual behavior, and discussed how researchers could increase the veracity and believability of their work using said methods.	Review	Matching procedures
[23]	Morgenstern	2013	Behavioural economics	Study where participants play a lottery or receive a sure amount. EEG recordings taken.	They hypothesized that real choices in a lottery choice paradigm should provoke a more careful comparison process, and that they would observe a higher level of cognitive control for real decisions because they are more salient.	Empirical	Cognitive control
[44]	Morkbak	2014	Behavioural economics	Choice experiment survey of preferences for apples using hypothetical and incentivized samples.	They explored potential differences in a choice experiment with real incentives and a hypothetical choice experiment by examining general preference structure, error variance, willingness-to-pay, and attribute non-attendance.	Empirical	Attribute non-attendance
[69]	Müller	2012	Behavioural economics	Choice set options, familiar brands at realistic price levels. The scenario was that the participant had found options of varying price levels for an item they need, and they must choose one (or buy one in binding setting).	They examined the compromise effect using an experimental design that incorporates some basic conditions of real purchases (e.g. unforced decisions) to investigate whether the compromise effect differs across choice settings (hypothetical or binding/real choice).	Empirical	Matching procedures; real consequences
[61]	Murphy	2005	Behavioural economics	Meta-analysis to assess bias in stated preference studies.	They attempt to evaluate the effect of different stated preference formats and other factors on the degree of hypothetical bias.	Review	High stakes rewards
[51]	Murphy	2004	Behavioural economics	Commentary about the need to better understand hypothetical bias and review of the literature.	Although the presence of this bias is well documented, its underlying causes are not fully understood. Consequently, this paper highlights the need for a better understanding of the causes of this bias, and argues that future experimental research should focus on this issue.	Review	Certainty; real consequences; self-image
[70]	Patil	2014	Moral reasoning	Text/virtual reality moral dilemmas. Four experimental settings (pitted the welfare of one individual against 2–3 individuals) and four control settings (controlled general differences across modalities).	Sought to examine how moral judgments translate into behavior.	Empirical	Space for mental simulation
[65]	Sacco	2017	Health behaviours	Systematic review that examined outcomes of actual (*N* = 6) or intended (*N* = 5) food purchasing decisions or consumption.	The purpose was to assess whether menu labelling influences the amount of calories ordered by children and adolescents (or parents on behalf of youth) in food outlets including restaurants and cafeterias.	Review	Matching procedures; real consequences
[59]	Scholl	2015	Neuroscience	Learning task, repeated choices between two options trying to minimize effort and maximise payoff. Three attributes of each choice: reward, effort, probability. Performed inside and outside an fMRI scanner.	They aimed to examine how contingencies are learned when an outcome has multiple components, only some of which should be learned.	Empirical	Salience of/ concern with the decision
[83]	Silva	2004	Behavioural psychology	Two studies: (1) hypothetical money rewards were devalued by delaying their occurrence, students made choices between smaller immediate and larger delayed rewards, and (2) real academic rewards were devalued by requiring more effort for larger rewards.	They examined whether higher and lower scoring students differed in their choices for outcomes devalued either by delay (Study 1) or by effort (Study 2).	Empirical	Matching procedures
[64]	Skoe	2002	Behavioural psychology	Asked respondents to rate the importance and difficulty of self-generated real moral dilemmas and three standardized hypothetical moral dilemmas.	The goal of the study was to examine the relationship of emotions with moral reasoning and the perceived importance of moral dilemmas.	Empirical	Personal relevance
[24]	Slovic	1969	Behavioural economics	Duplex gamble. Subjects faced 18 pairs with a probability range of winning and losing. Hypothetical and Real money groups.	The study was designed as a direct test of the hypothesis that individuals decide to maximize hypothetical gains, but cautiously decide to minimize chance of losing and amount to lose when real money is at stake.	Empirical	Risk aversion
[25]	Taylor	2013	Behavioural economics	Each subject made both real and hypothetical choices between gambles with varying probabilities and payoffs. Participants completed a cognitive test and questionnaire.	The study explored why some studies find that individuals are more tolerant of risk when making hypothetical choices than when making real choices.	Empirical	Cognitive ability
[42]	Teper	2015	Behavioural psychology	Two studies that test dissociation between behaviours and forecasts with a math test on which participants have a chance to cheat or to forecast cheating.	Their goal was to further clarify why people’s moral actions do not always match their predictions by varying the intensity of the affective experience.	Empirical	Emotional forecasting
[34]	Trevethan	1989	Behavioural psychology	Compared moral reasoning of incarcerated and non-incarcerated individuals along with antisocial personality scores.	They examined moral reasoning about hypothetical and real-life dilemmas from personal experience. They also examined stage of moral reasoning development and moral orientation.	Empirical	Personality traits
[85]	van Nieuwenhuijzen	2005	Social psychology	Addressed social problem solving in hypothetical (15 video vignettes) and real-life (fishing game). Compared how children with intellectual disabilities and externalizing behaviour problems respond and behave compared to kids without externalizing behaviour problems.	They investigated the relationships between responses provided in hypothetical problematic situations and responses occurring in similar real-life situations.	Empirical	Matching procedures
[49]	Verneau	2017	Behavioural economics	They used “food waste” as the target of participants’ implicit associations, and anti-waste certification as the target of participants’ willingness-to-pay.	They examined the effect of implicit associations on individuals’ behavior in hypothetical versus real auctions.	Empirical	Implicit associations
[26]	Vlaev	2012	Behavioural economics	Prisoner’s Dilemma. Manipulated reality (incentivized or not) and context (by putting medium cooperativeness round in a series of lower or higher cooperativeness games).	They examine whether hypothetical and real social behaviours are plagued by similar cognitive biases stemming from perceptual and action processes.	Empirical	Certainty
[47]	Xu	2016	Behavioural economics; neuroscience	Balloon analog risk task and EEG used to examine the effects of real and hypothetical monetary rewards on risk taking behavior among undergraduates.	They hypothesized that risk taking would show larger cerebral response to negative feedback during real monetary reward condition as compared to hypothetical reward condition.	Empirical	Risk aversion; real consequences
[48]	Xu	2018	Behavioural economics; neuroscience	They used event-related potential and measured brain responses to risk taking and decision making during the balloon analog risk task with large and small hypothetical or real monetary rewards.	They hypothesized that the response to real monetary reward would be stronger than the response to hypothetical monetary reward after negative feedback (money loss).	Empirical	Risk aversion

^a^Note: Direct quotes have been summarized for clarity and conciseness

## Abbreviations

PRISMA: Preferred Reporting Items for Systematic Reviews and Meta-Analyses
MeSH: Medical Subject Headings
